# Quantitative modeling of mortality patterns in dogs exposed to alpha particle emitting radionuclides: Insights from competing risks and causal inference machine learning

**DOI:** 10.1371/journal.pone.0328082

**Published:** 2025-07-21

**Authors:** Eric Wang, Igor Shuryak, David J. Brenner

**Affiliations:** Center for Radiological Research, Department of Radiation Oncology, Columbia University Irving Medical Center, New York, United States of America; Japan Atomic Energy Agency / Ibaraki University, JAPAN

## Abstract

This study employed state-of-the-art machine learning to evaluate the mortality effects of alpha-emitting radionuclides (^241^Am, ^249^Cf, ^252^Cf, ^238^Pu, ^239^Pu, ^224^Ra, ^226^Ra, ^228^Th) on 2,576 dogs, factoring in radioactivity levels, composition, administration method (injection or inhalation), and age at exposure. There were 972 cancer deaths, 599 non-cancer deaths, 789 deaths from many diseases (involving several diagnoses, including both cancer and non-cancer pathologies), and 216 deaths with uncertain causes. A Random Survival Forest model for overall mortality achieved concordance scores of 0.763 and 0.745 on training and testing data subsets, respectively. A model variant with competing risks was used to investigate mortality trends over time for different disease categories. It achieved concordances of 0.814 for cancer, 0.652 for non-cancer, and 0.778 for many diseases on training data, and 0.817 for cancer, 0.651 for non-cancer, and 0.780 for many diseases on testing data. All radionuclides exhibited radiation responses for cancer, with ^226^Ra and ^239^Pu showing the strongest effects. Some responses were non-linear, with indications of saturation or downturn at high treatment quantities. For non-cancer diseases, radiation responses were generally weaker and more variable. For the many diseases endpoint, ^238^Pu and ^239^Pu demonstrated the strongest response patterns, with 239Pu exhibiting greater lethality via inhalation compared to injection.. Using a Causal Forest model, which is designed to detect causal relationships rather than just associations, we investigated the causal impact of radioactivity on dog mortality, accounting for other variables. We found a significant (p < 2 × 10^−16^) negative average causal effect of −1,375 days per log_10_ radioactivity unit on survival time. This study improves current knowledge of cancer and non-cancer mortality patterns from densely-ionizing radiation in mammals by using machine learning to analyze combined historical data on dogs exposed to different radionuclides, modeling multiple variables, nonlinear dependencies, and causal relationships.

## Introduction

Human data on the health effects of densely-ionizing radiations, such as alpha particles, neutrons, and heavy ions, is limited, making the use of animal models essential. Investigating radiation exposure and its health outcomes in non-human mammalian species is crucial for understanding the associated risks. Traditionally, rodent models have been the primary choice for such studies, offering valuable insights. However, to fully investigate the complexities of radiation-induced health risks and to understand inter-species differences, a broader range of animal studies is necessary. Understanding inter-species differences is critical because anatomical, physiological, and metabolic variations can profoundly influence how radiation is distributed, absorbed, and biologically processed within an organism. For example, differences in bone structure affect the deposition and retention of bone-seeking radionuclides like ^226^Ra and ^239^Pu, leading to varying radiation dose distributions and subsequent cancer risks. Additionally, species-specific DNA repair mechanisms and cellular responses to ionizing radiation play a key role in determining the likelihood and type of radiation-induced diseases. By studying larger animals such as dogs, which exhibit closer anatomical similarities to humans compared to rodents, researchers can better predict the human health risks associated with densely ionizing radiation exposure. This understanding is particularly important for refining radiation protection guidelines and improving the translational relevance of animal model findings to human populations. During the 20th century, dogs (mainly of the beagle breed) were frequently used in radiation research in both the USA and Europe. These studies involved exposing the dogs to various types of radiation, including both external radiation sources and internal exposure from radionuclides [1–9].

Although such data were collected under ethical standards of the time, these historical experiments may not always conform to modern ethical standards. Consequently, the use of historical data allows present day researchers to gain insights into radiobiological effects without conducting new large scale animal experiments. Revisiting these datasets with modern analytical methods provides an opportunity to honor the original sacrifice by extracting additional value. This approach not only avoids new large animal studies but also reduces the ethical burden associated with future research by leveraging pre-existing resources. In addition, dog populations continue to be the subjects of radiation research up to the present time, for example the free-roaming dog populations that inhabit the Chernobyl nuclear power plant accident zone for decades since the accident and continue to be chronically exposed to ionizing radiation [10].

Previous research on irradiated dogs has documented many types of diseases. For example, there was a lot of work performed on investigating the oncogenic potential of radionuclides like ^226^Ra and ^90^Sr in inducing bone sarcoma in beagle dogs, highlighting similarities in disease pathology between canines and humans, which are critical for translational medicine [1,2]. Furthermore, these studies have shown that the distribution of alpha-emitters within bone structures plays a pivotal role in cancer development [3], demonstrating how radiation damage can be intricately linked to anatomical features [4]. For instance, Hahn *et al.* (1999) detailed how inhaled alpha and beta particles in dogs led to different types of neoplasms, with alpha particles inducing more aggressive forms of lung cancer compared to beta particles [5]. These studies have extensively investigated the effects of inhaled ^239^Pu and ^144^Ce in beagle dogs, providing valuable insights into the effects of these radionuclides [5]. They demonstrated that while both types of radionuclides could induce lung cancer, the mechanisms and histological types of tumors varied significantly. Specifically, ^239^Pu was found to be more effective at lower doses compared to ^144^Ce, which required higher doses to achieve similar oncogenic effects. Furthermore, these studies highlighted the importance of considering both the type and mode of exposure when assessing radiological health risks.

There were also several investigations into genetic changes in canine lung neoplasms following exposure to ^239^Pu. They have revealed a scarcity of mutations in crucial oncogenes such as p53, erbB-2, and K-ras [6,11]. This suggests alternative mechanisms of carcinogenesis, which may involve epigenetic modifications or other regulatory disruptions, rather than direct genetic alterations [7,12]. The study by Lloyd *et al.* (1994) also supports this, highlighting the complexity of dose-response relationships in irradiated dogs [8,9].

Important international efforts were undertaken to combine the data from these diverse investigations into a single database to facilitate broader conclusions from the wealth of accumulated experimental data and identify general trends, which can translate from dogs to humans. Much of the available data on radiation research in dogs (and other mammalian species) was collected by the European Radiobiological Archives (ERA), a collaborative initiative by the Federal Office for Radiation Protection in Germany and the University of Cambridge, England [13]. Supported by the 6th Framework Programme of the European Commission, the ERA aims to consolidate and provide access to data from long-term animal studies that are vital for advancing current understanding of radiobiological impacts [14].

Large-scale experiments involving tens of thousands of animals, which are stored in ERA, are unlikely ever to be conducted again due to financial and ethical reasons. By reanalyzing historical datasets, such as those curated by the ERA, we maximize the value of past studies while minimizing the need for future large-scale animal experiments. This aligns with the ethical principle of reducing animal use and ensures that the original studies contribute to modern advancements in science and public health.

In this study, we accessed the ERA dog database. This data set was quite diverse in terms of administered radionuclide types (Table 1). Most irradiated dogs were treated with ^239^Pu or ^226^Ra, but seven other isotopes were also used on substantial numbers of animals. This diversity of exposure types is informative for assessing the influence of specific isotopes on the health outcomes observed. The characteristics of the isotopes are summarized in Table 1.

**Table 1:** Radionuclide characteristics and numbers of exposed dogs in the analyzed data.

Radionuclide	Half-life	Radiation types emitted	Sites of greatest accumulation	Number of dogs exposed
^241^Am	432.2 years	α, γ	bones, liver, muscles [15]	117
^249^Cf	351 years	α	liver, bones	30
^252^Cf	2.645 years	n, α	liver, bones [16]	30
^238^Pu	87.7 years	α	bones, liver [17]	144
^239^Pu	24,110 years	α	bones, liver [5]	757
^224^Ra	3.63 days	α	bones	110
^226^Ra	1,600 years	α	bones, liver, and muscles [18]	456
^228^Th	1.91 years	α	bones [15]	81

One of the important purposes of such databases is to facilitate re-analyses of historical data sets using constantly evolving newer methods, particularly machine learning (ML) techniques. The use of state of the art ML methods like Random Survival Forests (RSF) with competing risks and Causal Forests (CF) for causal inference can significantly enhance the analysis of older experimental data on dogs and other animals exposed to radiation. These methods are capable of handling complex interactions and non-linear relationships between variables in high-dimensional data, such as combined data from numerous historical experiments. This capability makes it feasible to combine results from different studies into a single analysis, increasing the statistical power and allowing for more robust and detailed conclusions. By uncovering complex interactions, these methods can provide new insights that might not be evident from a traditional analysis. For example, in the context of radiation studies, an individual might be subject to more than one cause of death (*e.g.,* cancer and non-cancer diseases). RSF can account for these competing risks in a flexible manner without strict assumptions about relationships between variables and dose response shapes. Furthermore, CF can extend inferences beyond the realm of correlations and associations into the domain of causal inference. It can estimate not only the average effect of radiation across the population, but also heterogeneous radiation effects at the individual level, which can help understand the differential effects of radiation exposure on different subgroups of animals based on their characteristics (*e.g.,* age, type of radionuclide).

We previously utilized such state of the art ML methods to analyze large collections of historical mouse experiments involving densely ionizing radiation, revealing complex dose-response relationships and underscoring the importance of considering competing risks when assessing mortality outcomes [19]. In the current study, we employed ML techniques to evaluate the effects of various alpha-particle-emitting radionuclides in another rich and unique data set collection provided by ERA on mortality of beagle dogs. To our knowledge, this is the first ML-based investigation of these dog data sets. We believe that the study usefully contributes to current scientific understanding of the health risks associated with long-term exposure to densely ionizing radiations in mammals.

## Methods

### Data collection and preparation

This study utilized a comprehensive dataset of 2,576 dogs from the European Radiobiological Archives (ERA), including records of individuals exposed to alpha particle emitting radioisotopes and unexposed controls. Detailed preprocessing was conducted using the R programming language. Details of the preprocessing steps are provided in Supplementary Methods, and a brief description is given below. The dataset is available upon request from the ERA, which is hosted by the Bundesamt für Strahlenschutz (BfS) in Germany, subject to approval by their data governance committee.

The dataset included radioactivity dose, exposure duration, and age at exposure. However, it did not include information on sex, and thus this variable was not considered in the analysis. Mortality was classified into three groups based on the pathology type reported at the time of death: cancer (972 cases), non-cancer (599 cases), and many diseases (789 cases, which included a variety of diagnoses, both cancerous and non-cancerous). This classification was used for the subsequent competing risks analysis, allowing for distinct evaluations of mortality hazards and cumulative incidence functions for different causes. To evaluate the predictive performance and generalizability of the competing risk models, the dataset was randomly divided 75%:25% into training and testing subsets.

During preprocessing, variables such as Group ID and Age at Death (the age at death in days) were excluded as they were identifiers or target variables unsuitable for use as predictors. Additionally, variables like Treatment Unit and Treatment Application were excluded after being encoded into more informative representations (*e.g.*, Injection and Inhalation). There was no missing data – all retained individuals had complete records for the variables of interest.

Continuous variables were left un-normalized, in their original units, since the tree-based models used in this analysis are scale-invariant. However, for causal analysis, Treatment Quantity, which represents the amount of radioactivity administered to the dog (in kBq/kg body weight), was log-transformed to reduce skewness and stabilize variance. Categorical variables were processed using one-hot encoding, with separate binary variables created for each radionuclide (*e.g.*, ^241^Am, ^239^Pu) and treatment method (Injection, Inhalation, Control). A custom classification function was applied to Pathology Type (which described the pathologies detected at death) to assign each dog to one of four categories: cancer, non-cancer, many diseases (multiple or uncertain pathologies), or censored observations (*e.g.*, accidental or unknown deaths).

This process retained key variables directly associated with radiation exposure, such as dose and duration, alongside demographic factors like age. The final set of predictors included radiation-related factors such as ^241^Am, ^249^Cf, ^252^Cf, ^238^Pu, ^239^Pu, ^224^Ra, ^226^Ra, and ^228^Th, as well as treatment-related variables like Treatment Quantity, Treatment Age, Injection, Inhalation, and Control.

### Modeling approaches

The analysis framework encompassed three distinct approaches outlined below, with details provided in Supplementary Methods:

*Random Survival Forest (RSF) for Prediction of Deaths from All Causes*: A RSF model from the grf R package was applied to predict all-cause mortality, using Age at Death as the failure time. Hyperparameters of the model were tuned, and importance scores for the predictor variables were calculated, providing insights into the relative significance of each predictor.

*Competing Risks RSF for Prediction of Different Disease Categories Separately*: For the competing risks analysis, we used the randomForestSRC R package. This allowed us to analyze cancer and non-cancer outcomes separately, which was essential for understanding the distinct mortality trends from different disease types while considering multiple possible causes of death. To achieve optimal splitting during tree construction, we employed a modified weighted log-rank rule, known as logrankCR. This rule, based on Gray’s test, calculates weighted log-rank statistics for each competing event type. It then combines these statistics into a single measure to identify the best possible splits. The key advantage of this method is that it considers all competing events simultaneously, unlike the standard log-rank test that would treat other events as censored. To ensure robust generalization, we assessed overfitting through independent test-set validation, where RSF’s C-index remained stable between training and testing (e.g., 0.814 vs. 0.817 for cancer, 0.652 vs. 0.651 for non-cancer, and 0.778 vs. 0.780 for many diseases). This suggests that the model generalizes well without substantial overfitting.

In the context of competing risks, two main concepts are used to understand and model risk over time:

- Cause Specific Cumulative Hazard Function (CSCHF): This function represents the cumulative rate at which a specific type of event occurs over time. Essentially, this function captures the risk of an event occurring at any given time, considering that other events might compete with it. The CSCHF is particularly useful when the analysis focuses on determining factors for event-specific risk. The generalized log-rank test, used in this context, is based on the weighted difference of the Nelson-Aalen event-specific cumulative hazard estimates in the daughter nodes, which tests for equality of the event-specific hazard. For example, imagine studying cancer-related deaths: the CSCHF represents the total “burden” of cancer mortality risk up to a given time, calculated by integrating the instantaneous hazard rate (the likelihood of dying from cancer at each moment). This function is foundational for identifying factors that influence event-specific risks.
- Cumulative Incidence Function (CIF): The CIF is derived from the CSCHF and represents the probability that a particular event will occur by a certain time. It provides a way to account for competing risks in a survival model. Unlike the CSCHF, which focuses on the rate of events, the CIF calculates the probability of an event happening by a certain time while considering that other competing events might also occur. So, while the CSCHF quantifies cumulative risk, the CIF answers a more intuitive question: “What is the probability someone will die from cancer by age 70, given they might die from something else first?”

These functions allow to model the risk landscape for different event types more comprehensively, providing a clearer understanding of how competing risks influence overall outcomes. For example, while the CSCHF reflects the instantaneous risk of a specific event (such as cancer-related death) at any point in time, the CIF gives the probability that this event has occurred by a certain age, taking into account the possibility that other events (such as non-cancer deaths) may occur first.

For competing risks, the concordance score (C-index) evaluates whether subjects who experience the event of interest earlier have higher predicted probabilities (CIF values) than those who experience it later or not at all. C-index calculation involves the following definitions. Comparable Pairs: We compare pairs of subjects where at least one experiences the event of interest (e.g., cancer death). Pairs where both experience competing events (e.g., heart disease deaths) are excluded, as they provide no information about cancer-specific risk ordering. Handling Competing Events: If Subject A dies from cancer at 5 years, and Subject B dies from heart disease at 3 years, Subject B is treated as censored at 3 years for cancer-specific risk. The model checks if Subject A’s predicted CIF at 5 years exceeds Subject B’s predicted CIF at 3 years. For example, if a high-risk patient (predicted CIF = 0.8 at 5 years) dies from cancer earlier than a low-risk patient (predicted CIF = 0.2 at 3 years), the pair is concordant—the model correctly ranks their risks.

Beyond concordance indices, further evaluation of the competing risks RSF model’s performance was undertaken to measure its ability to differentiate between specific disease categories (*e.g.*, cancer vs. non-cancer mortality). We calculated sensitivity and specificity measures for each disease category at age 5000 days. Sensitivity was defined as the proportion of correctly identified events of a specific type (*e.g.*, cancer) among all true events of that type, while specificity measured the proportion of correctly identified non-events for that category.

A traditional Cox proportional hazards (CoxPH) model was also fitted to the data using the same predictor variables as the RSF model to provide a baseline comparison. The Schoenfeld residual test was used to evaluate the proportional hazards assumption, and survival curves were generated to visualize mortality risks. The CoxPH model’s performance was assessed using the concordance index (C-index) on training and testing subsets. To evaluate the advantages of RSF, we compared its performance to two widely used parametric models: Cox proportional hazards and Fine-Gray competing risks models. While these models are well-established, they rely on restrictive assumptions about proportional hazards and linearity, which may not hold in complex radiobiological datasets.

- Cox proportional hazards model: The Cox model’s proportional hazards assumption was violated for multiple key predictors (p-values < 0.01 for Inhalation, ^226^Ra, ^239^Pu, and ^228^Th), suggesting that the estimated hazard ratios may be biased. Furthermore, the Cox model’s C-index dropped from 0.726 (training) to 0.281 (testing), indicating severe overfitting and poor generalizability.
- Fine-Gray competing risks model: The Fine-Gray model performed moderately well for cancer mortality (C-index = 0.782 at 5261 days) but failed for non-cancer mortality (C-index = 0.5 at 5261 days), which was lower than RSF’s non-cancer prediction (C-index = 0.651).
- RSF provided the most robust generalization across all mortality categories, particularly for non-cancer diseases, where it captured complex nonlinear dependencies that Fine-Gray failed to model. The final competing risks RSF model achieved C-index values of 0.817 (cancer), 0.651 (non-cancer), and 0.780 (many diseases) on the test set, compared to 0.814, 0.652, and 0.778 on the training set. These results justify the use of machine learning models over traditional survival approaches in this dataset.

*Causal Analysis Using Causal Forest (CF) to Explore the Effect of Radioactivity on Survival*: A causal analysis was performed on irradiated individuals with known causes of death using a causal forest approach. This method estimated individual causal effects of radiation treatment on survival times, focusing on non-censored observations.

### Hyperparameter tuning for RSF and CF Models

For the RSF model, hyperparameter tuning was performed to optimize num.trees (number of trees) and mtry (number of variables randomly selected at each tree split). We conducted a grid search over the following ranges: num.trees = [30, 40, 50, 75, 100, 125, 150] and mtry = [6 8 10 12 14 ]. For each combination, we calculated the concordance index on the training data using out-of-bag (OOB) predictions and selected the parameters with the highest concordance. This tuning process ensured robust performance without overfitting.

For the Causal Forest (CF) model, we utilized the grf package’s built-in tuning procedures. A forest with 5,000 trees was trained, with honesty enabled to avoid information leakage between splits and parameter tuning conducted over a subset of the forest (tune.num.trees = 1,000). Model calibration was tested using the test_calibration function in the grf R package. A coefficient of 1 for mean forest prediction indicates the accuracy of the model’s average predictions. Meanwhile, a coefficient of 1 for differential forest prediction, along with its significant p-value, indicates the model’s effectiveness in capturing heterogeneity in the treatment effects, suggesting variability in how different subjects respond to similar treatments.

### SHAP value analysis for interpretability of the competing risks RSF model

SHAP (SHapley Additive exPlanations) values are a method to explain the output of any machine learning model [20]. They are based on cooperative game theory and assign an importance value to each feature in a model, representing its contribution to the model’s output. SHAP values help researchers understand how each feature influences final model predictions, the contribution of each feature compared to others, and the model’s reliance on the interaction between features. These values can also account for non-linear and non-monotonic relationships between variables. SHAP values are additive, meaning that the contribution of each feature to the final prediction can be computed independently and then summed.

However, it is important to note that SHAP values can be potentially misleading if the predictor variables are strongly correlated with each other. In this study, the fastshap R package was used to calculate approximate Monte Carlo-based SHAP values for the model-predicted CSCHF at age 5000 days. The fastshap package assumes feature independence by default in its approximation of SHAP values. This is a trade-off for computational efficiency and simplicity. However, if the dataset has strong feature correlations, interpret results should be done cautiously. Consequently, we evaluated feature correlations and considered this information in interpreting SHAP values.

We confirmed SHAP value consistency by comparing the sum of SHAP values across predictors to the difference between individual predictions and the average model prediction. This consistency check ensures reliable interpretation of variable contributions. Further visualizations, including dependence plots and correlation matrices, provided nuanced insights into interactions between predictors, especially for radionuclides with significant mortality impacts. Additional details of the SHAP value computation and visualization methods are provided in the Supplementary Methods.

### Ethics Statement

This study involved a retrospective analysis of publicly available, fully anonymized data from historical experiments conducted on dogs. As the data are de-identified and intended for machine learning analysis, no new animal experiments were conducted, and no additional ethical approval was required. The original experiments followed ethical guidelines in place at the time, under the oversight of the relevant Institutional Animal Care and Use Committees (IACUCs). Specific details regarding anesthesia, euthanasia, or other handling methods were not available in the dataset.

## Results

### Overview of variables in the data set

**Fig 1:**
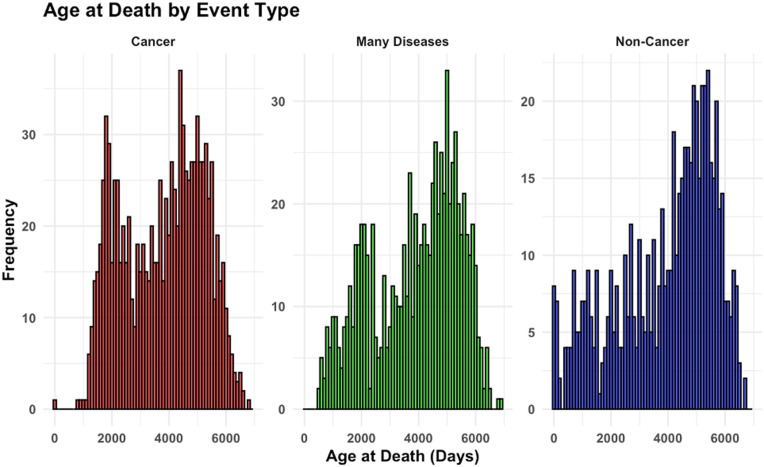
Distributions of age at death (shown on the x-axis) for three distinct event types among the dogs: cancer (red), many diseases (green) and non-cancer (blue). Each panel represents one event type, with the y-axis indicating the frequency of deaths within each category.

In the data set there were 972 individuals with cancer identified as the cause of death, 599 with non-cancer, 789 with many diseases (*i.e.,* several identified diagnoses, often including cancer and non-cancer pathologies), and 216 with missing or uncertain cause of death (coded as censored in the analysis). Summary statistics for the most important variables in the data set are outlined in Table 2, highlighting differences between different disease categories in terms of treatment age (*i.e.,* age when radioactivity was administered), age at death, and administered radioactivity amount. A visualization of the frequency distributions of age at death for different disease categories are shown in Fig 1. Although the median and mean ages at death were relatively similar for dogs across the three disease categories (cancer, non-cancer, and many diseases), the histograms in Fig 1 indicate some differences in the age distribution patterns. For example, a peak in deaths from cancer and many disease categories occurred at early ages (around 2000 days), whereas no such prominent peak was observed for non-cancer diseases. However, for all disease categories the main mortality peaks occurred at ages around 5000 days.

**Table 2:** Summary statistics for the most important variables in the data set. Treatment Age = dog age (in days) at the start of radioactivity administration. Negative values indicate prenatal exposure for pregnant females. Age at Death = age (in days) at death. Treatment Quantity = administered radioactivity amount (kBq/kg body weight).

Variable	Minimum	25^th^ percentile	Median	Mean	75^th^ percentile	Maximum
	**Cancer** (972 total dogs)					
Treatment Age	−63	400	400	412	435	1700
Age at Death	0	2502	4088	3821	5001	6754
Treatment Quantity	0	0.0074	2.22	38.1	33.3	370
	**Non-Cancer** (599 total dogs)					
Treatment Age	−63	400	400	381	400	1700
Age at Death	0	2733	4462	3967	5306	6700
Treatment Quantity	0	0	0.185	24.4	2.37	370
	**Many Diseases** (789 total dogs)					
Treatment Age	−52	90	400	289	400	1700
Age at Death	500	2535	4201	3879	5128	6913
Treatment Quantity	0	0	0.36	4.08	4.07	111

### Random survival forest (RSF) for prediction of deaths from all causes

To optimize the predictive accuracy and robustness of our RSF model, we tuned the model’s hyperparameters, and the best combination turned out to be 50 trees and 14 features (mtry) considered at each split in the decision trees that make up the forest. The model demonstrated strong concordance scores, with 0.763 (standard error, SE = 0.006) on the training set and 0.745 (SE = 0.013) on the testing set. The small decrease in performance from training to testing suggests good generalization and absence of serious overfitting.

The variable importance scores (VIMPs) of various features in the RSF were quantitatively assessed, with Treatment Quantity emerging as the most influential predictor, followed by ^224^Ra and Control (an indicator variable for no radiation exposure). The complete list of feature importance scores, outlined in Table 3, provides a detailed insight into the predictors that most significantly affect survival outcomes across all causes of death in our RSF model. Among the different radionuclides, ^224^Ra and ^239^Pu achieved the highest importance scores. The mode of radioactivity administration (injection vs. inhalation) was also a notable factor, with Injection and Inhalation ranking among the top predictors. However, it is important to consider that only ^238^Pu and 239Pu were administered by inhalation (with ^239^Pu also given via injection), whereas all other radionuclides were administered exclusively through injection.

**Table 3:** Variable importance (VIMP) scores for RSF for all causes of death, sorted from highest to lowest.

Feature	Importance
Treatment Quantity	0.758
^224^Ra	0.053
Control	0.043
Treatment Age	0.040
^239^Pu	0.029
Injection	0.024
Inhalation	0.021
^228^Th	0.019
^226^Ra	0.010
^241^Am	0.001
^238^Pu	0.001
^239^Cf	0.000
^252^Cf	0.000

### Competing risks RSF for prediction of different disease categories

In addition to the analysis of all deaths combined, we implemented the competing risks RSF model version to predict different disease outcomes separately. The competing risks model, with an optimal number of 100 trees, demonstrated robust performance, as evidenced by the out-of-bag (OOB) performance errors, which were closely aligned between the training and testing datasets. On the training data, the concordance scores for cancer, non-cancer, and many diseases categories were 0.814, 0.652, and 0.778, respectively. On the testing data, the corresponding concordance scores were 0.817, 0.651, and 0.780. This near parity in performance between the test and training datasets suggests good strong mode generalization and the absence of overfitting. This consistent performance across both datasets suggests that our modeling approach effectively captures the underlying patterns without tailoring the fit too specifically to the training data—a common challenge in predictive modeling. These results highlight the utility of our model in reliably predicting different mortality outcomes across multiple disease categories.

The RSF model also demonstrated high sensitivity (75.8%) for cancer outcomes, reflecting its strong performance in detecting cancer-related deaths, with a corresponding specificity of 74.3%. For non-cancer outcomes, sensitivity was 11.8%, while specificity remained high at 96.0%, indicating the model struggles to identify non-cancer cases but effectively excludes them. The many-diseases category exhibited very high sensitivity (97.5%) and decent specificity (78.2%), suggesting that the model reliably identifies many-diseases cases but occasionally misclassifies them as other outcomes. These results highlight that the model performs best for identifying cancer outcomes compared to the more heterogeneous non-cancer and many-diseases categories.

To provide a performance benchmark for the competing risks RSF model, we compared its performance to a traditional Cox proportional hazards (CoxPH) model. The CoxPH model was fitted using the same predictor variables, with age at death as the time-to-event outcome and cancer, non-cancer, and many diseases as the competing risk categories. The Schoenfeld residual test indicated that while some predictors satisfied the proportional hazards assumption (e.g., ^239^Pu p = 0.029, ^228^Th p = 0.006, and Injection p = 0.468), others exhibited significant deviations. Notably, Inhalation (p = 0.007), ^226^Ra (p = 0.0006), and Treatment Quantity (p < 2 × 10 ⁻ ¹⁶) showed strong violations, highlighting potential limitations in the CoxPH model’s applicability to this dataset.

The CoxPH model achieved a C-index of 0.726 on the training set, but this dropped dramatically to 0.281 on the testing set. This stark decline highlights severe overfitting and the poor generalizability of the CoxPH model to unseen data. In contrast, the RSF model maintained stable generalization, as described above. This performance gap underscores the advantages of machine learning methods, particularly in capturing complex, non-linear relationships and interactions in high-dimensional datasets. These findings illustrate the limitations of traditional survival analysis methods like CoxPH, especially in datasets with intricate dependencies, time-varying effects, and non-proportional hazards, as observed in this radiobiology study.

The feature importance scores in the competing risks RSF model varied noticeably between the different disease categories, offering insights into the differential impact of each predictor on each disease type. For example, Table 4 shows that Treatment Quantity was more influential in predicting cancer outcomes and many diseases (importance scores of 0.329 and 0.228, respectively) compared to non-cancer outcomes (0.042). Other variables such as Treatment Age, ^238^Pu, and ^228^Th also showed notable differences in their importance between cancer and non-cancer predictions, highlighting the unique contributions of these factors to each disease outcome. This detailed analysis of variable importance not only enhances our understanding of the predictors but also underscores the utility of the competing risks approach in disentangling the effects of various factors on distinct types of disease outcomes.

**Table 4:** Variable importance (VIMP) scores for competing risks RSF for cancer, non-cancer, and many diseases. They are sorted from highest to lowest for the cancer category.

Feature	Importance		
	**Cancer**	**Non-cancer**	**Many Diseases**
Treatment Quantity	0.329	0.042	0.228
Injection	0.143	0.092	0.098
Treatment Age	0.118	0.044	0.244
^224^Ra	0.071	−0.012	0.036
Inhalation	0.051	0.041	0.083
^228^Th	0.042	0.005	0.036
226Ra	0.036	0.001	0.024
Control	0.031	0.010	0.036
^239^Pu	0.021	0.027	0.012
^238^Pu	0.015	0.003	0.017
^241^Am	0.009	−0.005	0.006
^252^Cf	0.006	0.004	0.003

**Fig 2:**
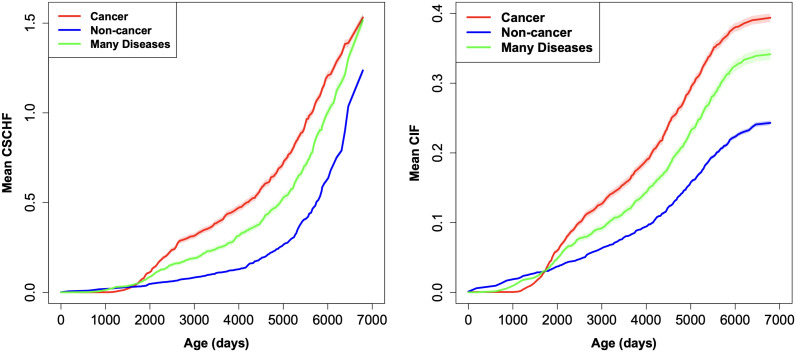
CSCHF and CIF plots for cancer (red), non-cancer (blue), and many diseases (green), generated by the competing risks RSF model fit to the training data. Shaded regions represent 95% confidence intervals for both CSCHF and CIF, quantifying the uncertainty in predictions across time.

Visualization of the competing risks RSF results was carried out through the CSCHF and the CIF functions, which indicate the risk landscape for mortality from each disease type over time (Fig 2). Inspection of the mean values of these functions calculated over all trees in the RSF (Fig 2) reveals the dominant nature of cancer as a cause of mortality in the studied canine population, especially at ages >2000 days. The CSCHFs for all three disease categories continued to increase with age even at the oldest ages, but CIFs expectedly began to saturate at ages >6000 days because there were few surviving dogs remaining at such ages. To quantify the uncertainty in these CIF predictions, we calculated 95% confidence intervals for both CSCHF and CIF. These confidence intervals (Fig 2) were narrow for most time points, indicating the reliability of predictions for each cause of death. Additionally, the non-overlapping intervals across disease categories underscore the model’s discriminatory power.

**Fig 3:**
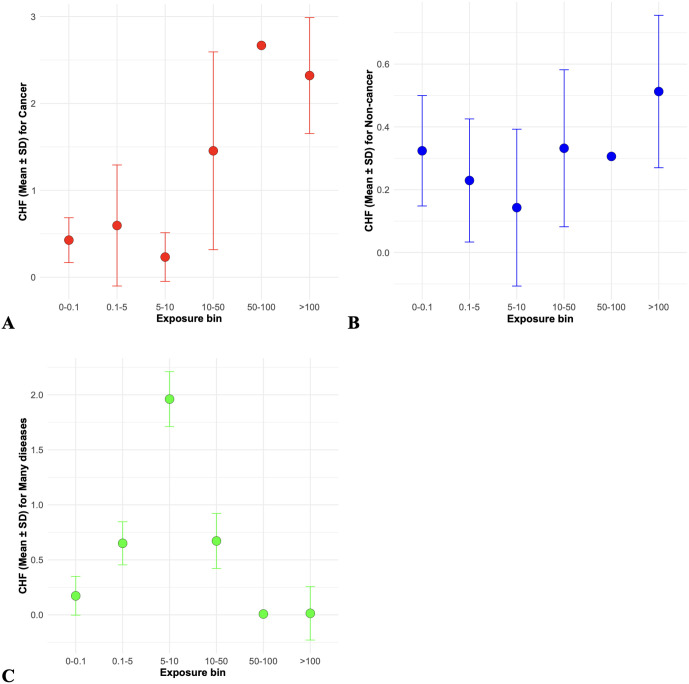
Distributions of the CSCHF at age 5000 days for A = cancer (red), B = non-cancer (blue), and C = many diseases (green) by exposure bin (based on Treatment Quantity). Exposure bins were based on Treatment Quantity and represent the following ranges: 0–0.1, 0.1–1.5, 5–10, 10–50, 50–100, and >100 kBq/kg. These bins were selected to capture a broad spectrum of exposure levels while balancing the number of individuals in each group. Circles represent the mean CSCHF values for each bin, and error bars show the standard deviations within each bin.

A visualization of how mean CSCHF values for different disease categories vary with Treatment Quantity is shown in Fig 3. The dose response trend is most clearly visible for cancer, with the highest mean CSCHF values at 5000 days occurring in the highest exposure groups. In contrast, the mean CSCHF for the many diseases category peaks at intermediate exposure levels and appears to decrease at the highest exposures, while the CSCHF for non-cancer outcomes shows a more complex trend, appearing to decrease initially and then rise again (Fig 3). These complex patterns suggest that those individuals exposed to the largest radioactivity amounts had high cancer or non-cancer death hazards, whereas their hazard of having many disease diagnoses at death was not as high. By comparison, at intermediate radioactivity levels many dogs tended to have numerous diseases diagnosed at death.

**Fig 4:**
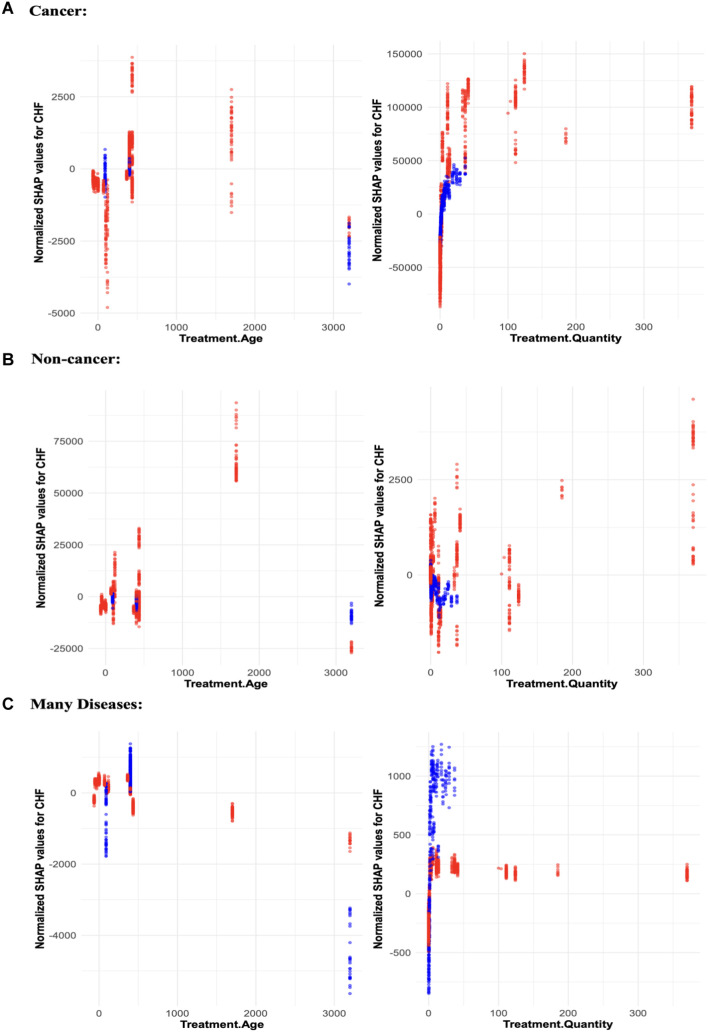
Visualizations of how the normalized SHAP values of each continuous feature in the competing risks RSF model change as function of the feature values for injection (red) and inhalation (blue). The top two panels are for cancer, the middle two are for non-cancer, and the bottom two are for many diseases. The SHAP values were calculated for CSCHF at age 5000 days.

**Fig 5:**
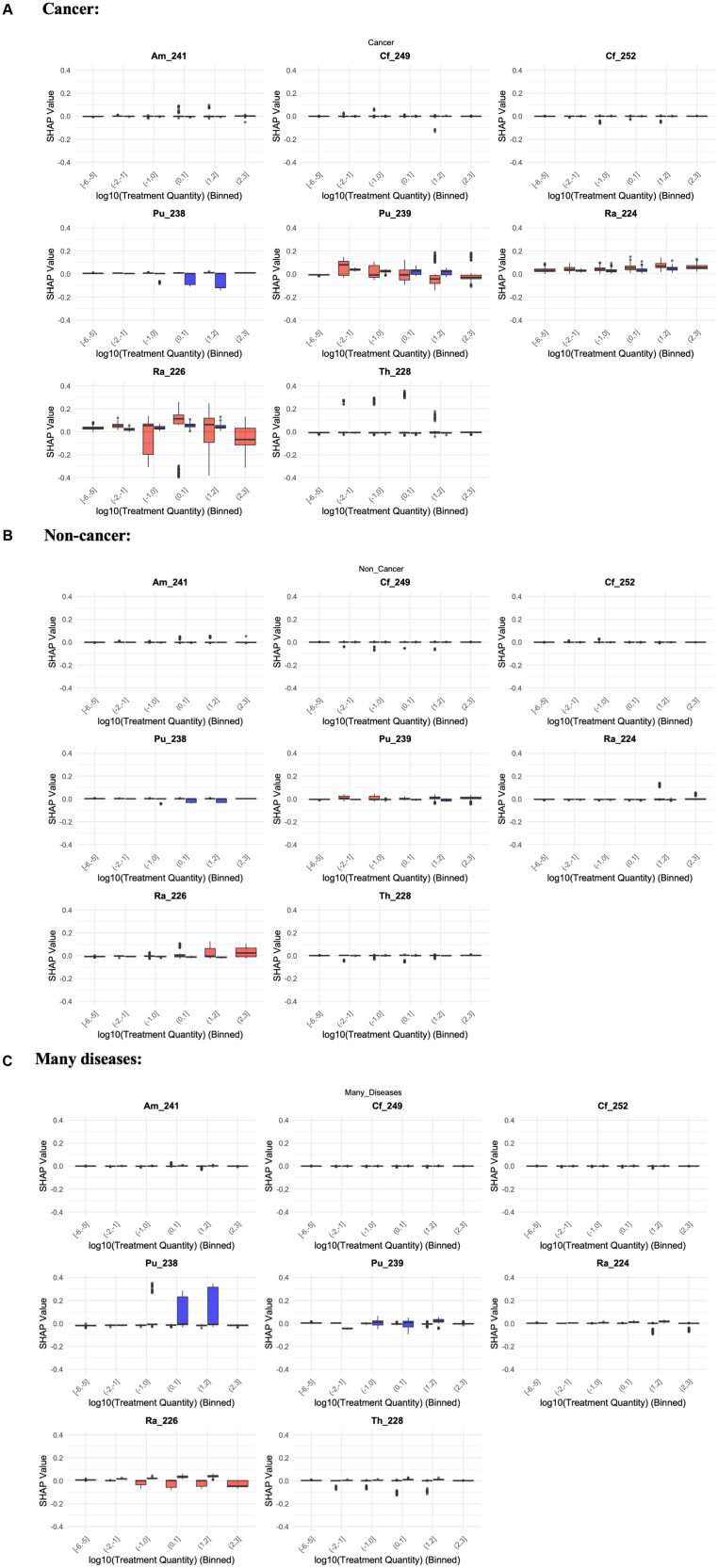
Boxplots of SHAP values for individual radionuclides, displayed on the y-axis, are plotted against log-transformed treatment quantity, displayed on the x-axis. Each panel corresponds to a specific radionuclide, allowing for a comparison of SHAP value patterns across different radionuclides. The plots are divided into three disease categories: Cancer (top group of panels), Non-Cancer (middle group), and Many Diseases (bottom group). Bars are color-coded based on the treatment method, with red representing injection and blue representing inhalation. The units on the x and y axes in all panels are consistent to facilitate comparisons of radiation response patterns across radionuclides and disease categories.

SHAP value analysis quantified the impact of each predictor on mortality risks from each disease category (Figs 4-5, Table 5). The SHAP trends for all radionuclides are summarized in Fig 4, and those for each radionuclide separately are shown in Fig 5. The trends for the effects of Treatment Quantity in the SHAP value plots for all radionuclides combined (Fig 4) continue to mirror those in the mean CSCHF summary plots (Fig 3). For many diseases, a peak at intermediate exposure levels is largely attributable to radioactivity administration by inhalation, rather than by injection. It is important to note that inhalation was used only for administering ^238^Pu and ^239^Pu (with ^239^Pu also administered via injection), while all other radionuclides were administered solely by injection.

**Table 5:** Median SHAP values (calculated for CSCHF at age 5000 days) for binary features in the competing risks RSF model for different disease categories. The features are sorted in decreasing order by absolute value for the cancer outcomes.

Feature	Cancer	Non-cancer	Many diseases
Injection	−0.161	−0.052	0.185
Inhalation	0.072	0.022	−0.105
^226^Ra	0.036	−0.007	0.005
^224^Ra	0.036	−0.004	0.001
Control	0.016	0.000	−0.007
^228^Th	−0.008	0.001	0.001
^239^Pu	−0.008	−0.003	0.001
^238^Pu	0.005	0.002	−0.016
^241^Am	−0.001	−0.001	0.000
^252^Cf	0.000	0.000	0.000
^249^Cf	0.000	0.000	0.000

The SHAP value analysis for separate radionuclides (Fig 5) revealed distinct patterns across the different radionuclides and treatment quantities for cancer, non-cancer, and many diseases. For cancer outcomes, ^226^Ra and ^238^Pu exhibited the strongest positive SHAP values as Treatment Quantity increased, indicating a clear association between higher treatment quantities and increased cancer mortality risks. The SHAP values for ^226^Ra were consistently positive across the treatment range, reinforcing the strong link between these radionuclides and cancer mortality. For cancer outcomes, ^239^Pu exhibited strong SHAP values, with no clear difference in mortality risk between inhalation and injection.

For other radionuclides like ^249^Cf, ^228^Th, and ^241^Am, the SHAP values remained relatively stable with moderate increases at higher doses, showing a smaller but still positive effect on cancer mortality. In the non-cancer outcomes, the overall SHAP values remained much lower compared to cancer, with minimal variation across radionuclides and treatment quantities. Radionuclides such as ^226^Ra displayed slightly elevated SHAP values at the highest doses, but overall, the influence of treatment quantity on non-cancer mortality was far less pronounced. ^239^Pu, while showing some differentiation between inhalation and injection, had only a marginal impact on non-cancer outcomes.

For the many diseases category, the SHAP value trends were most pronounced for ^238^Pu and ^239^Pu. ^238^Pu showed a positive relationship between increasing Treatment Quantity and SHAP values, suggesting a strong association between higher treatment quantities and increased mortality from many diseases. Similarly, ^239^Pu showed strong SHAP values for many diseases, though the administration route (inhalation vs. injection) did not show a major difference in risk. ^226^Ra, though showing weaker associations compared to cancer outcomes, also contributed positively to the many diseases category.

Overall, this SHAP analysis highlights the critical role of Treatment Quantity and radionuclide type in determining mortality risks. Cancer outcomes are particularly sensitive to higher doses of ^226^Ra and ^238^Pu, while many diseases are also influenced by these radionuclides, especially through inhalation. Non-cancer outcomes show much weaker associations, suggesting that these radionuclides predominantly affect cancer and multi-pathology outcomes. The clear distinction between inhalation and injection, especially for ^239^Pu, adds further complexity to the interpretation of SHAP values, with inhalation posing a greater risk in both cancer and many diseases categories.

The effects of Treatment Age are also observable across all disease categories. Although these effects are not always monotonic, the oldest treatment ages are consistently associated with the lowest SHAP values, implying reduced risks as treatment age increases. The binary features summarized in Table 5 suggest that the methods of radioactivity administration—inhalation and injection—play a more substantial role in model predictions than differences between specific radionuclide types. For cancer and non-cancer outcomes, inhalation had positive SHAP values (indicating increased death hazards), while the trend was reversed for the many diseases category.

Statistical analysis was conducted to quantitatively assess the SHAP trends in Fig 5. Linear regression was performed to model to model the relationship between SHAP values from the competing risks RSF model as function of log-transformed treatment quantity for each radionuclide. Slopes and p-values for this analysis are reported in the supplementary materials (S1 Table in S1 File). For cancer endpoints, significant positive slopes (p-values < 0.05) were observed for ^241^Am, ^228^Th, and Treatment Age, confirming a strong radiation response. However, ^238^Pu, ^224^Ra, and ^226^Ra exhibited significant negative slopes, suggesting potential differences in their biological impact on cancer risk. For non-cancer endpoints, the trends were more variable. ^238^Pu, ^224^Ra, and ^226^Ra showed significant positive slopes, whereas ^249^Cf, ^239^Pu, and ^228^Th exhibited negative or non-significant associations. This mixed pattern indicates that some radionuclides may have a greater impact on non-cancer outcomes than others. For many diseases, most radionuclides displayed significant positive slopes, indicating a general increase in risk with exposure. However, ^224^Ra and ^249^Cf were notable exceptions, showing negative or non-significant associations. These findings highlight the complexity of radiation effects across different disease outcomes and suggest that certain radionuclides may influence mortality pathways differently (S1 Table in S1 File).

**Fig 6:**
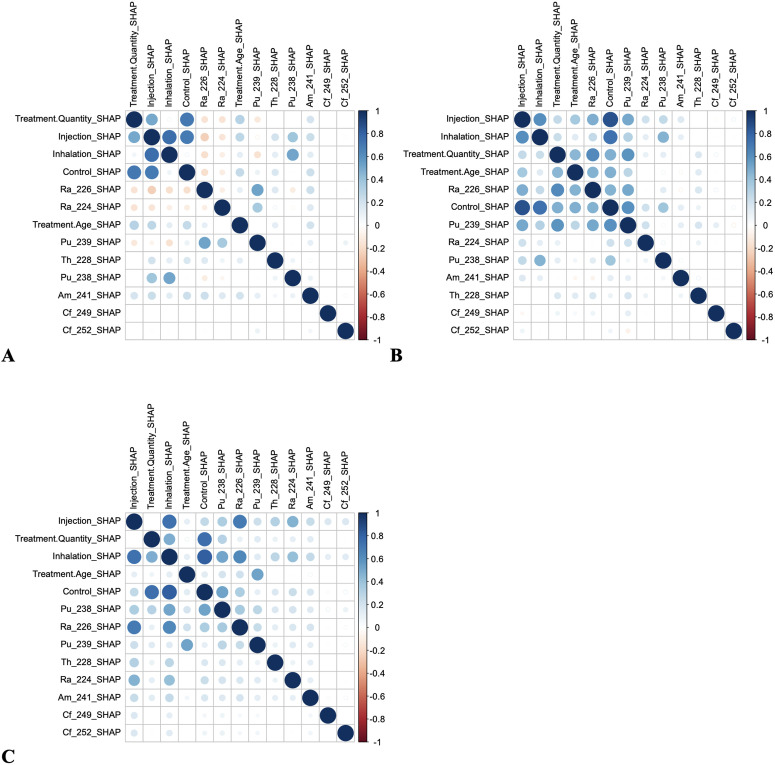
Pearson correlation matrices for SHAP values for the competing risks RSF model for different disease categories: A = cancer, B = non-cancer, and C = many diseases. The size of each circle represents the magnitude of the correlation coefficient, with larger circles indicating stronger correlations (closer to ±1). The color scale ranges from blue (positive correlations) to red (negative correlations), providing additional visual representation of the correlation direction and strength.

An additional angle to interpret the SHAP values is provided using Pearson correlation matrices (Fig 6). The strongest correlations were detected between the exposure variables like inhalation, injection and control, especially for cancer outcomes. For most other features, correlations of SHAP values were modest or low, < |0.5|. These results suggest that most of the variables in the RSF model did not contribute in a redundant manner, but each one had its own influence on model predictions.

### Causal analysis using causal forest (CF) to explore the effect of radioactivity on survival

We employed a CF model to examine how radioactivity can causally affect survival times among dogs who received some radioactivity and had known causes of death. There were 1,213 such individuals in the training set and 384 in the testing set. The causal (treatment) variable was log_10_-transformed Treatment Quantity and the outcome variable was Age at Death.

CF utilize “honest” estimation, which involves splitting the data into two subsets: one for constructing the tree structure and another for estimating treatment effects. This method reduces overfitting by ensuring that the same data points are not used for both determining splits and estimating outcomes. Unlike standard regression trees, CF employs a tree-splitting criterion that maximizes treatment effect heterogeneity between leaves. This means that the algorithm seeks to create splits that result in the most significant differences in estimated treatment effects across the resulting groups, which is crucial for identifying heterogeneous treatment responses. CF also ensure a minimum number of treated and untreated units (individuals) in each leaf to maintain estimation stability. This requirement helps to provide reliable estimates of treatment effects by ensuring that there are enough observations to support statistical inference within each subgroup.

CF aims to partition data into leaves, with the assumption that treatment effects are constant within each leaf. In other words, while treatment propensity scores and outcomes can vary across leaves, the treatment effect is considered homogeneous within each leaf. Within each leaf, CF performs a residual *vs*. residual regression, akin to double debiased machine learning (DML) [21,22]. In this approach, residuals from an outcome model (which excludes treatment, but includes covariates, which were ^241^Am, ^249^Cf, ^252^Cf, ^238^Pu, ^239^Pu, ^224^Ra, ^226^Ra, ^228^Th, Treatment Age, Injection, and Inhalation in this case) are regressed on residuals from a treatment model (which also excludes outcome). This method helps control for confounding factors while estimating treatment effects more accurately. In the grf R package, both sub-models are regression forests by default. The outcome was survival time in our case.

Each grf sub-model used 5000 trees and hyperparameter tuning [23]. The first model (treatment as function of covariates) had an R^2^ of 0.365 on training data, and the second model (outcome as function of covariates) had an even lower R^2^ of 0.081. These low performances are actually good for the causal analysis because they imply that the cause (radioactivity) or the outcome (survival time) are not well predicted based only on covariates, indicating a low risk of bias in the causal effect estimate [24].

To validate the reliability of the CF model, calibration tests were conducted, focusing on coefficients for mean forest prediction and differential forest prediction. The calibration results showed coefficients of 1.000 (SE = 0.020) and 0.996 (SE = 0.076) for mean and differential predictions, respectively, both significant at p < 2.2 × 10 ⁻ ¹⁶, reinforcing the reliability of our causal modeling approach.

The average treatment effect (ATE) estimated by the model for log_10_(Treatment Quantity) on Age at Death was strongly negative: −1375.2 (standard error 33.5, p < 2.2 × 10^-16^). This indicates that radioactivity, as expected, had a strong negative causal effect on dog survival time. On average, one log_10_ unit increase in Treatment Quantity reduced the age at death by 1375 days. It is important to note that while the causal forest analysis can provide strong evidence of a causal relationship, it does not definitively prove causality, for example because it relies on the assumption of no unobserved confounding (ignorability), which is not possible to definitively verify in real data. Thus, other factors which are not recorded in the analyzed data set could potentially be influencing the observed relationship. Therefore, these results should be interpreted in the context of the study design and subject to further validation.

**Fig 7:**
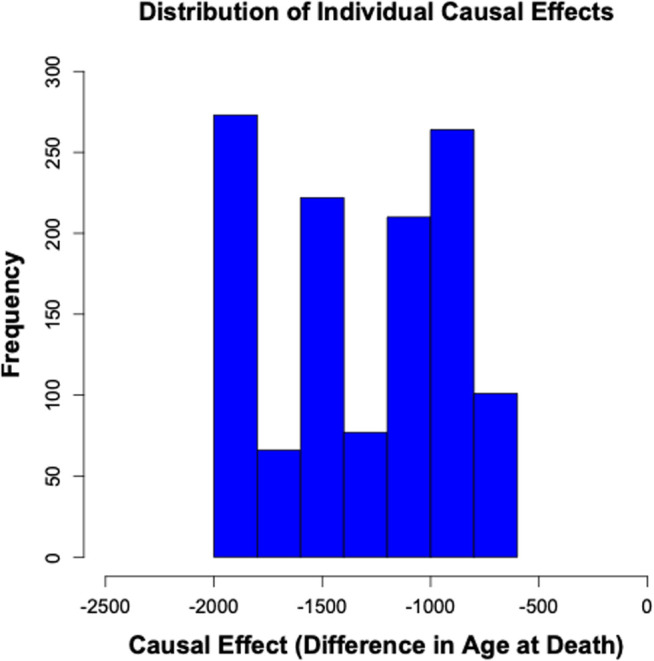
Distribution of individual causal effects on survival times. The histogram shows the frequency of various causal effect estimates for different dogs in the training data derived from the causal forest model.

A closer examination of the distribution of individual causal effects (Fig 7) helps to illustrate the considerable variability in the impact of radioactivity across different dogs within the study. Notably, this entire distribution was in the negative range (did not overlap zero), indicating that the estimated causal effect of radioactivity was detrimental for all individuals in the study.

The best linear projection (BLP) function was used to estimate a doubly robust linear approximation of the conditional average treatment effect (CATE) in the CF model, given a user-defined set of covariates. The double robustness property means that this approach provides valid estimates if either the treatment model or outcome model (but not necessarily both) is correctly specified. This method provides a way to intuitively interpret how the selected covariates influence the causal effect in an approximately linear manner. Table 6 presents the BLP focusing on the impact of treatment age and method (Injection *vs.* Inhalation) as selected covariates, and Table 7 presents the BLP focusing on the impact of different radionuclides. Notable findings from these BLP analyses include a significant negative effect for the intercept (Inhalation) and a positive effect for Injection (Table 6), suggesting that inhalation of radionuclides made the causal effect of radioactivity stronger (more negative), compared with injection. Treatment age showed a negative effect as well, suggesting that older age also made the causal effect more negative. The BLP results for different radionuclides (Table 7) indicated that ^238^Pu and ^228^Th had the most substantial negative impacts on the causal effect. ^224^Ra, ^252^Cf, and ^249^Cf showed the least negative effect, underscoring the varying influences of different radioactive substances (Table 7). This analysis helps in understanding the specific contributions of various radionuclides to the observed survival outcomes in the studied canine population.

**Table 6:** Best linear projection (BLP) of the conditional average treatment effect in the causal forest, focusing on impact of treatment age and method.

Variable	Estimate	Std. Error	t value	p-value
Intercept (Inhalation)	−1445.347	78.449	−18.424	< 2.2 × 10 ⁻ ^16^
Treatment Age	−0.795	0.116	−6.826	1.38 × 10 ⁻ ^11^
Injection	561.365	75.212	7.463	1.60 × 10 ⁻ ^13^

**Table 7:** Best linear projection (BLP) of the conditional average treatment effect in the causal forest, focusing on impact of different radionuclides.

	Estimate	Std. Error	t value	p-value
Intercept	−1666.03	107.76	−15.461	< 2.2 × 10 ⁻ ¹⁶
^241^Am	463.09	155.37	2.981	0.003
^249^Cf	652.87	257.25	2.538	0.011
^252^Cf	683.1	205.6	3.322	0.001
^238^Pu	−152.47	158.55	−0.962	0.336
^239^Pu	291.27	117.97	2.469	0.014
^224^Ra	819.5	250.64	3.27	0.001
^226^Ra	264.65	120.79	2.191	0.029

Future studies on the effects of radionuclides in other species could provide broader insights into interspecies variability. However, such efforts should also focus on reducing animal use by employing alternative models, using a non-canine species or models that do not experience pain at all, such as computational simulations or cellular systems, to ensure compliance with modern ethical principles of refinement and replacement.

**Fig 8:**
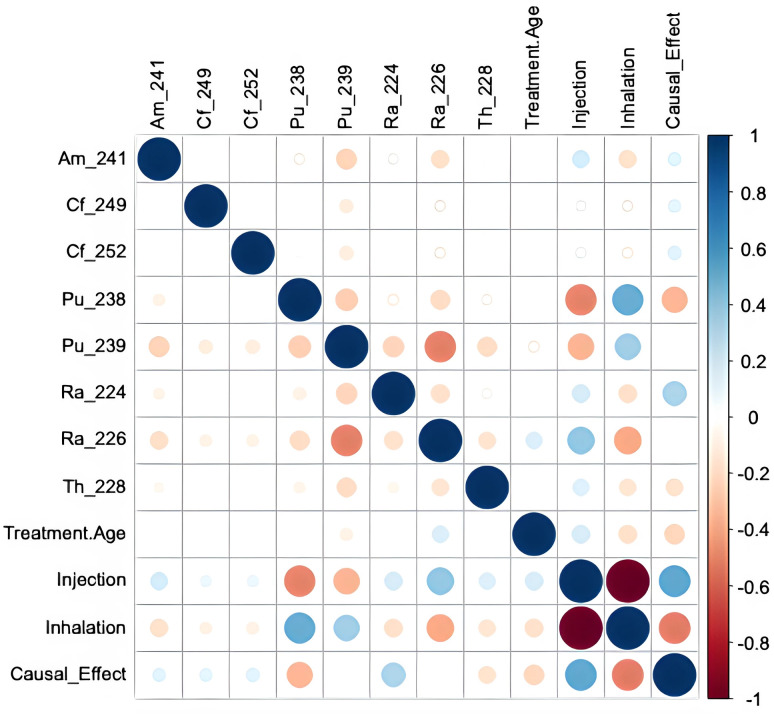
Pearson correlations of features with causal effects for all deaths generated by the causal forest on training data. The Causal Effect variable is the estimated effect magnitude. The size of each circle represents the magnitude of the correlation coefficient, with larger circles indicating stronger correlations (closer to ±1). The color scale ranges from blue (positive correlations) to red (negative correlations), providing additional visual representation of the correlation direction and strength.

Pearson correlation analysis was conducted to further assess the relationships between features and individual causal effects, revealing several notable associations (Fig 8). These visualizations indicate that the strongest negative correlations (suggesting strengthening of the causal effect) with the causal effect were found for inhalation and ^238^Pu, whereas injection and ^224^Ra had the opposite correlations. These findings highlight the importance of considering the route of radioactivity entry into the body, and the radionuclide types, when assessing the potential health risks.

## Discussion

This study employed machine learning (ML) methodologies to evaluate the impact of alpha-particle-emitting radionuclides (^241^Am, ^249^Cf, ^252^Cf, ^238^Pu, ^239^Pu, ^224^Ra, ^226^Ra, ^228^Th) on mortality in dogs by analyzing historical lifespan data from 2,588 individuals. To our knowledge, this is the first ML-based study on these data sets. The random survival forest (RSF) models for all deaths and specific disease categories generalized well from training to testing data portions without much loss of performance. In the competing risks RSF version, all radionuclides showed radiation responses for cancer, with some exhibiting non-linear patterns at high radioactivity levels. For non-cancer diseases, responses were less pronounced. In the many diseases category, ^226^Ra and ^239^Pu had the strongest responses, with no major difference in ^239^Pu lethality between inhalation and injection. A Causal Forest (CF) model revealed a significant negative causal effect of radioactivity on survival time, with ^238^Pu and ^228Th^ having the strongest effects.

Our findings on the collection of multiple irradiated dog data sets from ERA are broadly in line with those reported by previous studies, which looked at specific experimental subsets. For example, Hahn *et al.* (1999) reported that inhaled alpha particles from ^239^Pu induced pulmonary cancers [5]. Lloyd *et al.* (1994) provided further insights into the dose-response relationships for bone-seeking radionuclides, demonstrating that ^239^Pu had a significantly higher RBE for bone cancer induction compared to other radionuclides like ^226^Ra and ^241^Am [9]. These findings are qualitatively consistent with our observations of a potent carcinogenic effect of alpha-emitting radionuclides at relatively low doses. The detailed histopathological analyses from previous studies also align with our results, revealing various tumor types, including adenomas, adenocarcinomas, and squamous cell carcinomas, predominantly induced by alpha particles [9].

Since in this study we used ML methods on a large collection of dog irradiation data sets, rather than on specific data sets in isolation, our findings provide specific new insights into the effects of these radionuclides on mortality in a large and heterogeneous canine population. Our results showed that Treatment Quantity was the most critical predictor of mortality across all models, with cancer emerging as the dominant cause of death at higher exposure levels. Non-linear dose-response relationship for “many diseases” peaked at intermediate exposure levels and showed saturation or downturns at the highest doses, suggesting a more complex interplay of biological factors compared to cancer. These results highlight the importance of considering this heterogeneous category in analyses of radiobiological effects, as it reflects the cumulative impact of radionuclide exposures.

The causal analysis confirmed the detrimental effects of radioactivity on survival, with inhalation of radionuclides showing stronger impacts than injection. This highlights the importance of exposure route in determining health outcomes. The consistent identification of Treatment Quantity as the key factor across models underscores its central role in predicting mortality outcomes. This study also provides an example of combining predictive and causal inference approaches to understand both general trends and nuanced radiation effects. While the RSF models highlighted the importance of specific radionuclides like ^224^Ra and ^239^Pu, the CF model identified ^238^Pu and ^228^Th as having the strongest negative impacts. The inclusion of a CoxPH model as a traditional baseline demonstrates the limitations of parametric approaches in this context. While the CoxPH model provided insights into the proportional hazards and relative importance of predictors, its lower C-index on testing data compared to RSF and CF emphasizes the superior generalizability of machine learning methods.

While the RSF model demonstrated high performance for cancer mortality (C-index = 0.814), its predictive ability for non-cancer mortality was lower (C-index = 0.651). This result is expected given the greater heterogeneity of non-cancer deaths, which include cardiovascular, respiratory, and age-related conditions with multiple contributing risk factors. Many of these diseases may not have a direct radiation-related etiology, making prediction inherently more difficult. Despite these challenges, RSF still outperformed the Fine-Gray model for non-cancer mortality (C-index = 0.651 vs. 0.606), suggesting that machine learning models captured more complex risk interactions than traditional approaches. Future work should consider incorporating additional biomarkers or environmental factors to improve non-cancer mortality prediction.

The high C-index for cancer mortality (0.814) indicates that RSF provides clinically relevant risk stratification for radiation-induced cancers. This supports existing radiobiology findings showing strong dose-response relationships for bone-seeking radionuclides (e.g., Ra^226^, Pu^238^) in inducing malignancies. The lower C-index for non-cancer mortality (0.651) reflects the broader complexity of non-cancer diseases, which are influenced by multiple non-radiation-related factors. Despite this, RSF still outperformed Fine-Gray for non-cancer deaths, demonstrating its ability to model competing risk interactions. These results suggest that bone-seeking radionuclides like Ra^226^ and Pu^239^ are potent carcinogens, while inhalation-based exposure to Pu^238^ contributes to multi-organ damage, increasing mortality risk across disease categories. Future research should explore genetic markers of radiation sensitivity to refine risk assessment models and improve individual-level predictions.

We believe that this study provides new insights into the complex effects of alpha-emitting radionuclides on dog mortality, contributing to radiobiology. It uncovers intricate dose-response relationships, the impact of exposure methods, and the influences of different radionuclides, enhancing our understanding of radiation’s biological effects. Studies like this are important for refining radiation protection guidelines and risk assessment models, thereby improving public health and safety in radiation-prevalent environments such as occupational and accidental exposures and space exploration.

They also have relevance in the realm of radiation safety, healthcare, and experimental research. Specifically, stricter dose limits could be important for inhalation exposures involving radionuclides such as ^238^Pu and ^239^Pu, given their higher lethality compared to injection. These findings emphasize the importance of tailoring radiation protection guidelines to specific exposure scenarios. The observed relationship between Treatment Age and survival outcomes underscores the necessity of age-specific guidelines for medical radiation use. Such guidelines can help minimize adverse effects in older populations. Furthermore, the pronounced impact of inhalation on mortality highlights the need to prioritize respiratory protection measures in both medical and industrial settings to mitigate risks associated with inhaled radionuclides.

In experimental research, the detailed dose-response relationships and the distinction between cancer and non-cancer mortality patterns provide a valuable framework for designing studies on radiobiological effects in other species. The radionuclide-specific effects observed offer important insights that can guide future mechanistic studies and hypothesis generation. These findings illustrate the broader utility of combining machine learning and competing risks modeling to enhance the interpretation of complex radiobiological data.

In addition to its strengths, the study has some notable limitations. For example, the dataset used in this study, though extensive, is based on historical animal experiments that does not fully capture the diversity of dog breeds or real-world conditions. The absence of sex as a recorded variable restricts exploration of sex-specific radiation effects, which could provide valuable insights for human health risk assessment. However, species differences in anatomy, physiology, and DNA repair mechanisms influence radiation dose distribution and disease outcomes, underscoring the importance of expanding future research to investigate genetic factors and individual variations in radiation sensitivity across a wider range of species. Acknowledging these limitations is essential for effectively translating findings from animal models to human health risks.

Additionally, it is important to contextualize this study within modern ethical standards for animal research. While the data analyzed were collected decades ago under regulations in place at that time, modern approaches emphasize the principles of reduction, refinement, and replacement (3Rs) of animal use. Revisiting historical datasets with advanced methodologies reduces the need for future experiments and aligns with the ethical goals of minimizing harm to animals. Efforts to refine experimental protocols and develop non-animal alternatives, such as computational models or cellular systems, represent key priorities for future research in radiation biology.

In conclusion, the study has enhanced our understanding of the effects of alpha particle emitting radioisotopes on both cancer and non-cancer mortality in dogs. By using a comprehensive dataset and modern machine learning techniques, it has elucidated complex dose-response relationships and identified critical factors influencing mortality outcomes. Future research will build on these insights by leveraging existing datasets and integrating non-animal models, such as computational simulations and cellular systems, alongside genetic analyses to refine scientific understanding of radiation biology and enhance public health protection, in accordance with the principles of reduction, refinement, and replacement (3Rs) in animal research.

## Supporting information

S1 File Contains supplementary methods and the following supplementary table: S1 Table. (DOCX)
