# Supplementary material for: Quantitative modeling of mortality patterns in dogs exposed to alpha particle emitting radionuclides: Insights from competing risks and causal inference machine learning
Source: PLoS One. 2025 Jul 21;20(7):e0328082. doi: 10.1371/journal.pone.0328082 (PMC12279112; doi:10.1371/journal.pone.0328082)
Supplement: S1 File — (DOCX) [file pone.0328082.s001.docx]

**Supplementary Methods**

**Data Preprocessing and Analysis**

Several data preprocessing tasks were performed on the dataset. Initially, all character columns were converted to factors. The percentage of missing values (NA) in each column was calculated, and rows with missing values were subsequently dropped. The distribution of the lengths of diagnosis strings in the Pathology Type column was examined. This involved calculating the number of characters in each diagnosis string, converting these lengths to their logarithmic (base 10) values, and creating a histogram of the log-transformed string lengths. Additionally, summary statistics for the log-transformed string lengths were printed, and the 70th percentile of the original string lengths was calculated to be approximately 100 characters, which was then used as a cutoff for further analysis.

A custom function was defined to classify diagnoses into four categories: censored (0), cancer (1), non-cancer (2), and many diseases/uncertain diagnoses (3). The function first checked for NA values, assigning them to the censored category. It then checked for keywords associated with non-natural death, assigning these cases to the censored category as well. Diagnoses with string lengths exceeding 100 characters were classified as many diseases/uncertain diagnoses. Keywords indicating uncertain diagnoses were assigned accordingly. Finally, the function looked for cancer-related keywords and classified these cases as cancer; if no cancer keywords were found, the diagnosis was classified as non-cancer. The function was applied to the Pathology Type column, creating a new column, Cancer Status, to store the classification results.

Additional steps were performed to prepare a cleaned dataset version for further analysis. Relevant variables were selected from the original dataset, including specific isotopes (*e.g.*, Am_241, Cf_249), treatment-related information (*e.g.*, Treatment Quantity, Treatment Unit, Treatment Application), and age-related data (*e.g.*, Treatment Age, Age at death). The Treatment Application variable was cleaned by creating three new binary variables: Injection, Inhalation, and Control. A new variable, Death_event, was created, setting its value to 1 for all rows. The data were then split into training and testing sets using a random seed for reproducibility. Finally, the Es_253 variable was removed from both the training and testing sets, as this isotope was only used on six individuals.

**Detailed Modeling Steps**

- **Random Survival Forest (RSF) for Prediction of Deaths from All Causes:** Predictor variables were separated from the outcomes, and the model's hyperparameters were tuned by defining ranges for num.trees and mtry values. Concordance scores were computed for both the training and test sets.
- **Competing Risks RSF for Prediction of Different Disease Categories Separately:** Relevant variables were selected, and the competing risks RSF model was fit using the training set. Predictions were made on the training and test sets, and performance error values were documented.
- **Causal Analysis Using Causal Forest to Explore the Effect of Radioactivity on Survival:** The Treatment Quantity variable was log-transformed to improve scaling. Regression forest models were built to predict the treatment and outcome variables, and a causal forest model was constructed to estimate the treatment effects. Calibration tests and the calculation of the average treatment effect were performed to validate the model.

**SHAP Value Computation and Visualization**

Helper functions were created to calculate the medians and absolute medians of the SHAP values, and for plotting SHAP values. Correlations for the SHAP values were calculated and visualized using the corrplot function.

**Supplementary Tables**

**Table S1.** Statistical analysis of SHAP value trends for different disease outcomes. Linear regression was performed to model to model the relationship between SHAP values from the competing risks RSF model as function of log-transformed treatment quantity for each radionuclide. Slopes and p-values for this analysis are reported in this table.

| **Radionuclide** | **Cancer** | | **Non-Cancer** | | **Many Diseases_** | |
| --- | --- | --- | --- | --- | --- | --- |
|  | **Slope** | p-value | **Slope** | p-value | **Slope** | p-value |
| ^241^Am | 3.60E-04 | 9.29E-08 | 1.87E-04 | 1.57E-04 | -3.75E-05 | 1.31E-01 |
| ^249^Cf | 1.55E-05 | 7.35E-01 | -5.29E-05 | 1.82E-01 | -1.49E-05 | 8.87E-03 |
| ^252^Cf | -1.84E-05 | 5.70E-01 | 1.36E-05 | 3.28E-01 | -1.56E-05 | 5.27E-02 |
| ^238^Pu | -8.66E-04 | 1.34E-06 | -5.16E-04 | 1.75E-16 | 3.97E-03 | 4.83E-16 |
| ^239^Pu | 5.68E-04 | 1.04E-01 | 8.52E-04 | 3.16E-22 | -7.25E-04 | 5.36E-10 |
| ^224^Ra | -1.07E-02 | 2.40E-14 | 1.16E-03 | 1.77E-13 | -5.79E-04 | 3.85E-08 |
| ^226^Ra | -8.71E-03 | 9.80E-21 | 2.51E-03 | 3.15E-48 | -2.09E-03 | 1.99E-27 |
| ^228^Th | 1.31E-03 | 2.59E-04 | -1.44E-05 | 7.75E-01 | -3.65E-04 | 1.53E-03 |
